# Supplementary material for: Measuring and directing charge transfer in heterogenous catalysts
Source: Nat Commun. 2022 Jun 6;13:3253. doi: 10.1038/s41467-022-30923-2 (PMC9170698; doi:10.1038/s41467-022-30923-2)
Supplement: Supplementary file 1 — Supplementary Information [file 41467_2022_30923_MOESM1_ESM.pdf]

Supplementary Information

Measuring and Directing Charge Transfer in Heterogenous Catalysts

Authors: Michael J. Zachman[1]*, Victor Fung[1,2], Felipe Polo-Garzon[3], Shaohong Cao[1], Jisue Moon[3], Zhennan Huang[1], De-en Jiang[2], Zili Wu[3], and Miaofang Chi[1]*

Affiliations:

[1] Center for Nanophase Materials Sciences, Oak Ridge National Laboratory, Oak Ridge, TN 37831, USA.

[2] Department of Chemistry, University of California, Riverside, CA 92521, USA

[3] Chemical Sciences Division, Oak Ridge National Laboratory, Oak Ridge, TN 37831, USA

*Corresponding authors:

zachmanmj@ornl.gov

chim@ornl.gov

Supplementary Methods

Extracting the contribution of long-range interfacial charge transfer from 4D-STEM data. While CoM measurements now regularly provide atomic-scale information about materials, the contrast produced can be affected by many factors and careful interpretation is necessary to understand the origins of the signal. For example, local atomic-scale measurements are strongly affected by the shape of the probe[1], and both atomic-scale and long-range CoM measurements can be affected by non-uniform sample structure. It has been demonstrated, for instance, that by simply applying standard CoM analysis at a domain boundary in a polar-ordered material, the change in interface structure alone gives the appearance of locally bound charge even when none is present[2]. In this case, this is due to changes in the intensity within the central disk of the diffraction pattern that arise from features smaller than the probe[1]. The different crystal symmetry on either side of the domain wall thus produces varying contrast in the central disk as the probe is scanned across the boundary, which appears in the CoM signal. Charge distributions that result in long-range fields significantly larger than the probe, on the other hand, result approximately in a uniform disk shift (Supplementary Fig. 2)[3–6]. This shift is also encoded in the CoM signal if the disk edge is included in the calculation. Therefore, a method that isolates the disk shifts can aid in the interpretation of charge distributions that result in a long-range field, such as the case of charge transfer at the metal-oxide interfaces in heterogenous catalysts.

Since long range fields result in disk shifts, it is essential to separate the effect these have on the CoM from those due to contrast changes within the central disk, originating from atomic-scale structure, in order to verify their origin. Techniques for tracking changes at the edge of the central disk have been previously described[1,4] and allow features from long range fields to be extracted while minimizing effects from intensity variations within the disk. This suppresses contrast from both atomic-scale fields and effects from structure that appear within the central disk[2,4]. Here we modify the technique demonstrated by Krajnak et al.[4] for precise detection of subpixel shifts of the central disk to verify that the nanometer-scale CoM shifts observed in the main text originate from disk shifts.

Long range field mapping by disk shift tracking is typically performed using a nanometer-scale probe that results in separated diffraction disks. We chose to acquire the data in Fig. 2 and Fig. 4 of the main text with an Å-scale probe, however, which results in overlapping diffraction disks and enables simultaneous atomic-resolution imaging and minimization of the effect of probe shape on the measured charge distribution[1]. The effects of the long-range fields persist at the disk edge when acquired in this way, however, as shown in Supplementary Fig. 2. Though measurement of long range fields by an atomic-scale probe generally results in noisier results than measurement with a nanoscale probe, it has been demonstrated and discussed previously[5,7]. For thin samples, the diffracted disks are significantly weaker than the central beam, and a large difference in counts

within and outside of the central disk is therefore present, which enables the disk edge position to be tracked.

Supplementary Figure 6 shows the procedure used for isolating long range effects by central disk edge tracking. First, the diffraction data is first upscaled by factor of four using bilinear interpolation and a Gaussian filter with a two-pixel width applied (Supplementary Fig. 6b). An upper threshold value is then chosen and applied to each diffraction pattern, saturating the central disk for a uniform intensity across its surface (Supplementary Fig. 6c). To highlight the disk edge, the magnitude of the gradient of this pattern is calculated. This produces no contrast from within the disk due to the saturation, a sharp feature at the disk edge, and very weak features outside of the disk. To remove any additional contribution from these weak features, a lower threshold on the intensity values can also applied, leaving the ring identifying the disk edge (Supplementary Fig. 6d). Forgoing this second thresholding step for thin samples as shown here produces nearly identical results, however. Finally, a circular pattern with the same radius and position as the average central disk and a Gaussian radial profile is generated at the resolution of the upscaled diffraction data (Supplementary Fig. 6e). Each diffraction pattern processed as discussed above is then cross correlated with this circular pattern, and a Gaussian function is fit to the result. The position of this fit function at each probe position defines an array of central disk shifts with subpixel precision.

Since long-range fields result in disk shifts, an array of shifts generated as described above can be used to generate maps similar to those produced by CoM measurements, as shown in Supplementary Fig. 7. Comparing the results, the disk shift method suppresses the background of atomic-scale contrast, as anticipated, while preserving the longer-range features associated with the particle. Compared to the data shown in Fig. 4 of the main text, the charge density has a similar extended structure, but in this case no spatial filtering is necessary or used.

In addition to performing disk shift tracking on the large convergence angle data, smaller semiconvergence angles of 7 mrad and 2.5 mrad were also used to acquire data on the same set of particles for a comparison with more conventional parameters for tracking of long-range features. The results of disk shift tracking on these data sets is shown in Supplementary Fig. 7e-h. Qualitatively, the features produced match well those in the large convergence angle data. As expected, however, the resolution decreases with decreasing convergence angle, beginning to approach the size of the smallest particles by 2.5 mrad. By reproducing the long-range and suppressing the atomic-scale structure across multiple convergence angles, this confirms that the long-range CoM contrast arises primarily from a disk shift, as expected for a long-range field.

Supplementary Discussions

Effect of nanoparticle structure on CoM measurements.
In addition to confirming the source of the long-range CoM features by disk shift tracking, we also consider here the effect of the nanoparticle structure on CoM measurements. While the substrate structure is essentially constant in the system studied here and should have negligible effect on the measurements, the nanoparticle does introduce a perturbation to the overall structure, and inhomogeneous structure can affect the distribution of intensity within the central disk as mentioned above[2]. In addition, the added thickness of the sample due to the particle increases the local projected potential, thus leading to a beam deflection, the magnitude of which is proportional to the slope of the sample thickness[8]. This effect should be reduced with a larger probe size, however, since the proportion of the beam interacting with the sample thickness slope at a given probe position is smaller.

To explore this effect, we performed 4D-STEM simulations on the structure used for the DFT calculation in Fig. 3. This structure should have a larger effect on the probe than the structure in Fig. 5 since the addition of O to the perimeter somewhat flattens and broadens the particle, as seen in Supplementary Fig. 4, which reduces the slope of the projected potential increase, and hence generates a smaller beam deflection. To maximize the thickness effect, we also performed the simulations using the same microscope parameters that were used in acquiring the data shown in Fig. 2 and Fig. 4 of the main text, resulting in an Å-scale probe. The full three-dimensional potential was simulated with pyQSTEM based on the independent atom model[9] and the strong phase approximation was used to calculate the exit wavefunction after interaction with the sample[1,10]. As a consequence of using this model, no charge transfer was included, but the increased thickness of the sample at the nanoparticle location still resulted in an increased projected potential, which can be measured by 4D-STEM measurements, as shown in Supplementary Fig. 8. Although the extended structure is difficult to see in the atomic-resolution CoM and charge density maps, Gaussian filtering reveals that CoM measurements do give rise to a small apparent positive "charge" at the location of the nanoparticle. In addition to CoM, we also performed disk shift tracking measurements on this simulated data, which almost entirely removed atomic-scale contrast but did leave a positive feature on the scale of the particle, as in the case of the CoM. The disk deflections are much smaller than the CoM shifts, however, with the maximal value approximately 5x smaller than that of the CoM.

As opposed to the shape of the "charge" resulting from the CoM measurements, which is nearly flat across the particle, the "charge" measured by the disk shift tracking method is peaked in the center of the particle. This does not appear consistent with the experimental data, since generally more positive charge is located near the edges of the particles in Fig. 4, and disk shift tracking does not cause this intensity to shift and peak at the particle center (Supplementary Fig. 7). This effect should also be reduced for particles with oxygen at their perimeters, since this reduces the thickness

slope of the particle (Supplementary Fig. 4) and hence induced beam deflections, as mentioned above. This would suggest that the charge on the H-treated catalysts should be more positive than that on the O-treated catalysts, which is the opposite of what is observed. The larger particle in Fig. 2 does have a less negative value at its center, however, but we don't believe this is a likely a thickness effect either, since the effect shouldn't be localized to the center of the particle in these CoM measurements, according to our simulations. In addition, larger particles do not necessarily translate to a larger thickness-related "charge" since this is related to the thickness slope, and this would be necessary to overcome the increased charge transfer of the larger particle. Finally, we also performed simulations on the relaxed substrate structure alone, and we found that modifications of the support structure due to the particle produce negligible effects on the 4D data (Supplementary Fig. 8g-l). While we therefore conclude that the features observed are here mainly due to charge transfer, more weakly charged particles would require a more detailed analysis of these effects.

Support thickness estimation.
The thickness of the support layer was estimated by comparing simulated and experimental ADF data for a Au particle on an STO support. The structure used for the DFT calculations in the main text was again used in conjunction with pyQSTEM to generate the simulated ADF images. The image was then Gaussian filtered to allow a smooth line profile through the center of the particle to be generated. The same procedure was performed on the experimental ADF images generated from the 4D data using the same collection angles as the simulated data. The ratio of the known Au particle and STO support thicknesses from the simulated data was then compared to the ratio from the experimental data, and the known width-to-height ratio of ~2.4 for (111)-oriented Au particles on an STO (001) substrate[11] was utilized to estimate the thickness of the support as roughly 10 nm for the sample in Fig. 2 and <5 nm for the sample shown in Fig. 4.

Quantitative data interpretation.
While it is possible to assign quantitative values to the charges observed, it is not instructive to do so at this stage. The strength of beam deflections observed is a function of the width of Gaussian filter used and the spatial frequency of the features present. While we chose the smallest filter size that could remove the atomic-scale CoM information in the main text, some effect on the strength of the observed nm-scale charge transfer features would be present. Similarly, choice of convergence angle can affect the magnitude of the measured charge. As probe size approaches the feature size, such as in Supplementary Fig. 7, the strength of observed beam deflections is reduced. As a result, quantitative charge values provided would be dependent on these variables. In addition, changes in thickness of a material contribute to beam deflections, as shown in Supplementary Fig. 8. While we believe this contribution is not responsible for the majority of the qualitative features observed here, the contribution to a quantitative measurement would be non-negligible, shifting observed particle charges in the positive direction. Finally, imaging in projection through the catalyst and support makes observation of the regions of strongest charge accumulation at the particle-support interface challenging. In the future, it may be possible to measure these charges

using a perpendicular sample orientation (much like the DFT results shown in Fig. 5a,b), but this also significantly increases the difficulty of data interpretation since the substrate is no longer uniform beneath the particle, resulting in significant changes in sample thickness across the field of view and causing beam deflections as discussed above.

As a result, while it is possible to assign qualitative numbers to the charge transfer observed, we believe that results should currently be presented primarily through a qualitative matching of experimental and theoretical features, as performed in this manuscript.

Effect of alternate atomic configurations.

Charge transfer occurs from the support to the Au particle for both SrO- or TiO_2-terminated STO, with similar features, as shown in Supplementary Fig. 3. This originates from the work function of the Au nanoparticle, which was calculated to be ~5.0 eV regardless of STO termination and is higher than that of either of the STO surfaces, ~1.9 and ~4.5 eV for SrO- and TiO_2-terminations, respectively[12], which results in formation of a Schottky barrier at the interface between the materials[13]. While the SrO-terminated surface results overall in a somewhat larger charge transfer, as expected from the work functions, the main difference in the charge transfer features between the terminations is that the negative charge is more evenly spread over the particle in the TiO_2-terminated case, with increased charge transfer at the particle-support interface in the SrO-terminated case. It is worth noting, however, that while surface termination does not affect the direction of charge transfer in this system, other heterogenous metal-support catalyst systems have different work functions, and in certain cases the support surface termination may reverse the sign of charge on the metal particle, resulting in modified catalytic activity[14].

For DFT calculations emulating the O-treated sample, we considered atomic configurations altered from the pristine system through variations in quantity and position of oxygen atoms. Mainly, altered levels of oxygen vacancies in the support, additional oxygen atoms around the perimeter of the particle, and additional oxygen atoms covering the particle surface were considered. Some number of oxygen vacancies are likely to be present in the real system and increasing the number of vacancies should lead to a more negatively charged Au particle[15–17]. Since the DFT calculations of the pristine system showed the Au particle was already negatively charged, inducing oxygen vacancies in the support would not induce a positive charge, as our experiments show. Since including oxygen around the particle perimeter did induce a positive charge on the Au particle, as shown in Fig. 5, including oxygen atoms over the surface of the particle would likely induce an overall positive charge as well. However, we found that the charge transfer largely occurred locally near the oxygen atom positions, so placing oxygen over the surface of the particle would likely induce a positive charge over the entire particle surface and thus reduce the quality of the match with our experimental results. We therefore chose to focus on additional oxygen atoms around the perimeter of the Au particle. In the calculations shown in Fig. 5, twelve oxygen atoms were added near the perimeter of the particle, which consisted of 52 Au atoms, or about one oxygen atom per

four Au atoms. While this generated a highly positive charge on the particle, we found that even with four oxygen atoms, or one oxygen atom per thirteen Au atoms, the overall sign of the particle was still positive, with most of the positive charge still localized to the particle perimeter (Supplementary Fig. 9). In this case, a gap in the positive charge exists between one pair of oxygen atoms, though the overall form of the charge transfer is very similar to the case where twelve oxygen atoms were added.

Potential impact of secondary electron emission and plasmonic excitations.

While secondary electron emission can result in materials charging if electrons cannot be replenished quickly enough to compensate for those emitted, the additional material under the probe at the Au particles would likely increase the total emission rate. Any potential effects from secondary electron emission would therefore charge the material near the particle positively compared to the surrounding material. Since this is not consistent with the apparent negative charge on the H-treated Au-STO sample, we don't believe the effects of secondary electron emissions result in the overall features observed. In addition, plasmonic excitations produce transient fields around metal nanoparticles that could give rise to an apparent charge on the particles. Beam deflections due to these fields are observed for inelastically scattered electrons, however, which make up only a fraction of the overall signal. The resulting deflections would be exceedingly small for the size of particles observed in this work. Furthermore, these deflections would produce the appearance of a positive charge on the particle, which is again inconsistent with our observations of the H-treated Au-STO sample. As a result, we do not believe plasmonic excitations meaningfully contribute to the observed signals either.

Supplementary Figures

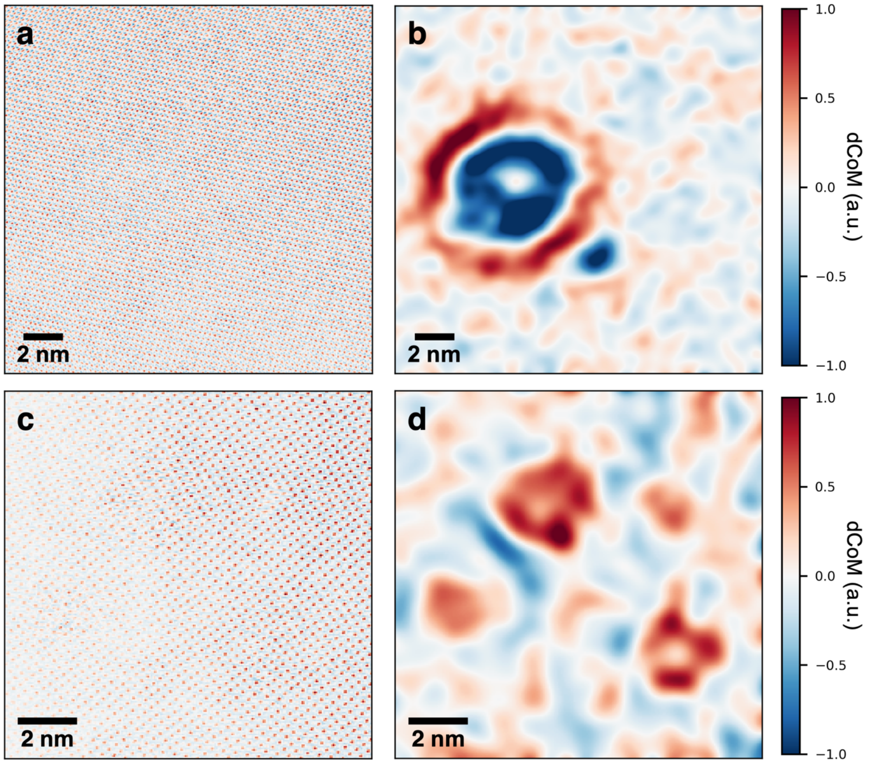

Supplementary Fig. 1 | Comparison of original and Gaussian-filtered inverted dCoM. a, Map showing the inverted divergence of the CoM shifts (dCoM) shown in Fig. 2 for the H-treated case, which is proportional to the charge density convolved with the probe shape for thin samples. **b**, Charge density map after application of a 4 Å Gaussian filter, which is approximately equal to the STO lattice constant, minimizing contrast from the support lattice and revealing a negative particle. **c**, **d**, Same as **a**, **b**, but for the O-treated case, showing positive nanoparticles.

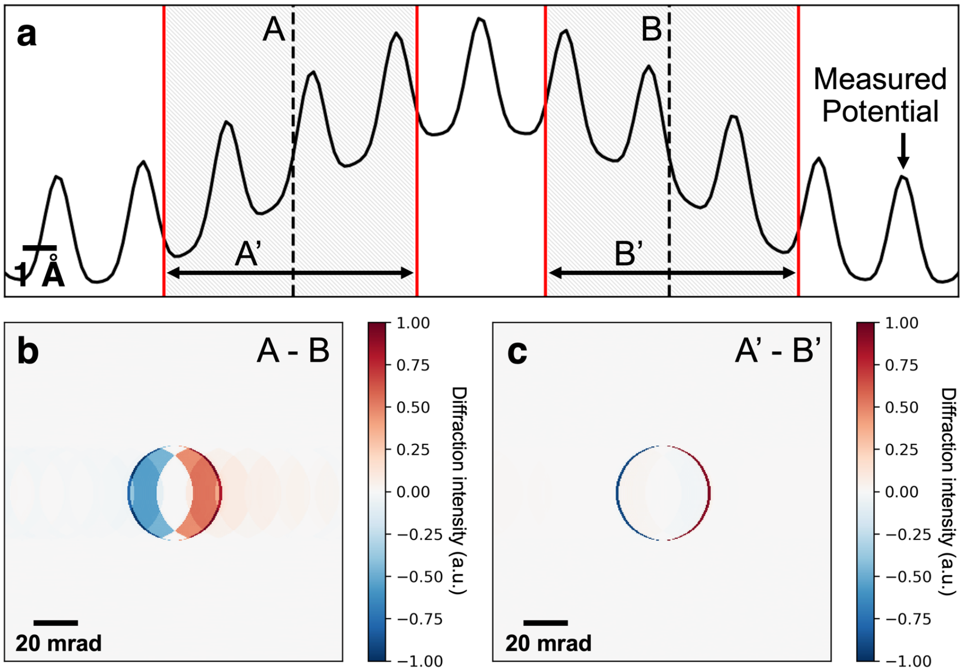

Supplementary Fig. 2 | Effects of a combined atomic-scale and nanometer-scale potential on electron diffraction using an Å-scale probe. a, Simulated one-dimensional profile of the combined potential measured by the CoM method used for the experimental data. The CoM encodes information about both the atomic and long-range potential. **b**, Difference in diffraction patterns from locations marked A and B in **a**. The effects of the long-range potential, namely the modified intensity at the disk edge, are seen even at individual probe positions. However, large intensity differences are also present inside the disk from the atomic potentials, due to interference between the central and diffracted beams. **c**, Difference in average diffraction between areas marked A' and B'. Averaging over unit cells minimizes contrast within the disk from the atomic-scale features while preserving the effects of the long-range potential.

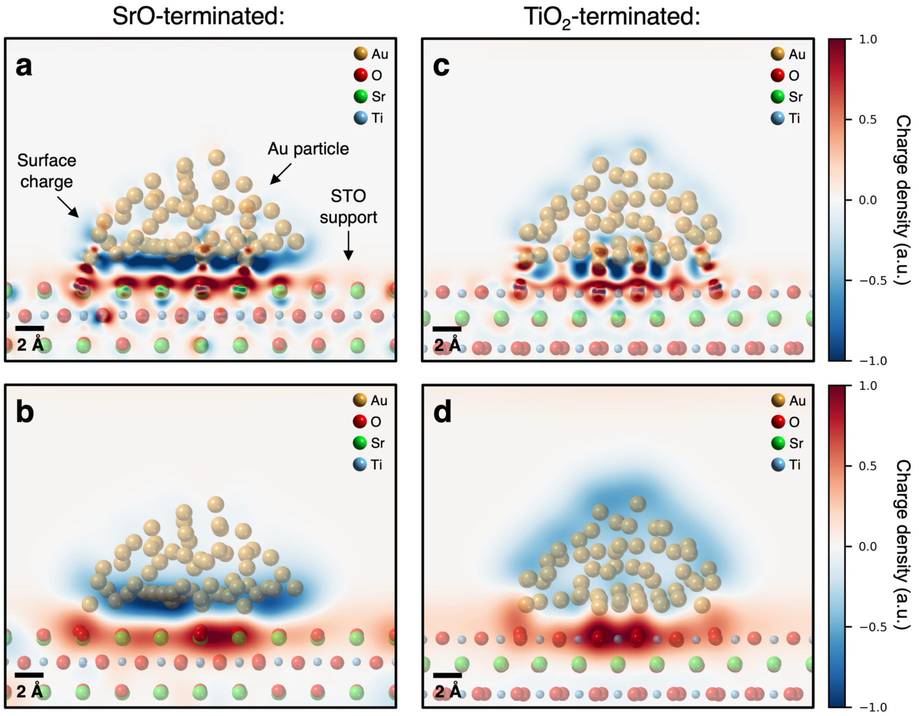

Supplementary Fig. 3 | Charge transfer at (111)-oriented Au nanoparticle/(001)-oriented TiO_2-terminated and SrO-terminated STO support interfaces, calculated by DFT. a, The SrO-terminated case is similar to the TiO_2-terminated case, **c**, with an overall negative particle and positive support, charge accumulation at the particle-support interface, and sharp positive features arising from strong Au-O interactions. **b**, **d**, A spatial filter like that used in Fig. 3 and Fig. 5 shows increased charge localized at the particle-support interface in the SrO-terminated case, and more evenly spread charge on the particle in the TiO_2 case.

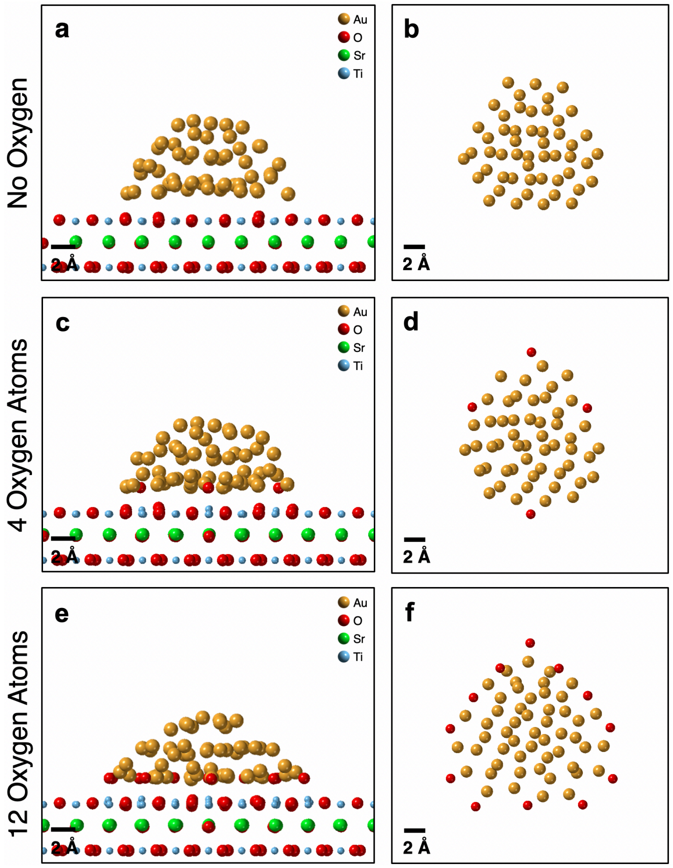

Supplementary Fig. 4 | Relaxed Au-STO structures from DFT calculations. **a**, **b**, Side and top-down views of the pristine Au particle on STO substrate including and not including the support atoms, respectively. **c**, **d**, Same as **a**, **b**, but for the structure with four oxygen atoms added to the perimeter of the particle. **e**, **f**, Same as **a**, **b**, but for the structure with twelve oxygen atoms added to the perimeter of the particle. In addition to changes in charge transfer, adding oxygen atoms near the perimeter of the particle somewhat reduces the height and increases the width of the particle, similar to a liquid droplet better wetting a surface.

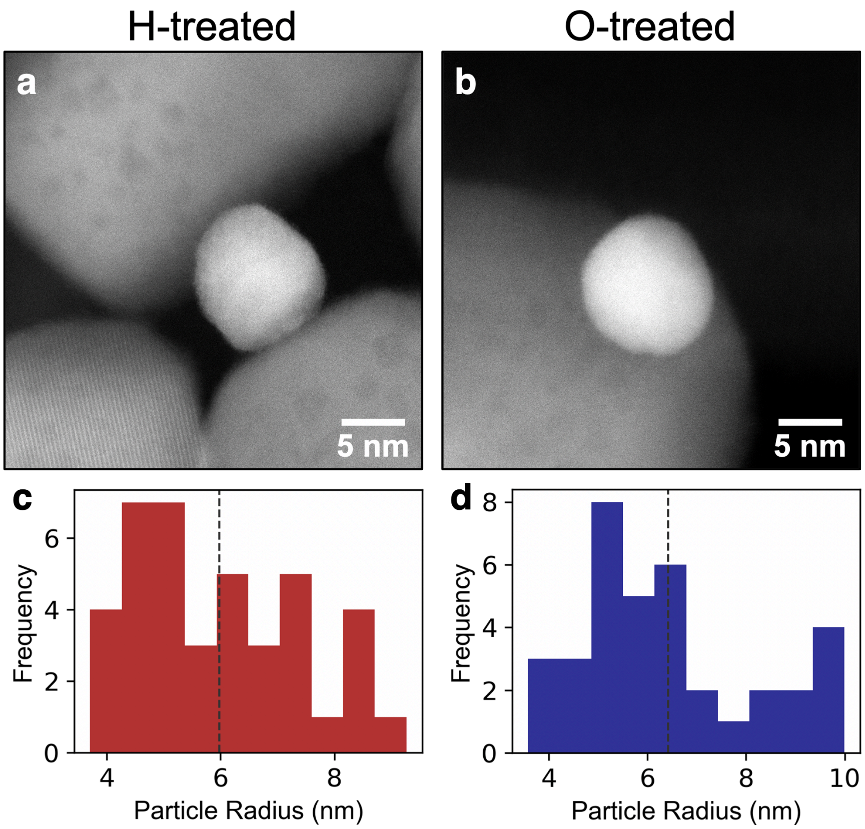

Supplementary Fig. 5 | Particle size measurements by HAADF-STEM of Au particles synthesized on an STO powder substrate, used for CO conversion performance tests. a, **b**, Example HAADF-STEM images of Au particles on a powder STO substrate after H- and O-treatment, respectively. **c**, **d**, Radius measurements for the H- and O-treated Au particles, respectively, with the mean particle radius indicated by the vertical dashed line. 40 H-treated particles and 36 O-treated particles were measured, with average radii of 6.0 ± 1.5 nm and 6.4 ± 1.8 nm, respectively, showing little difference in particle size between treatments.

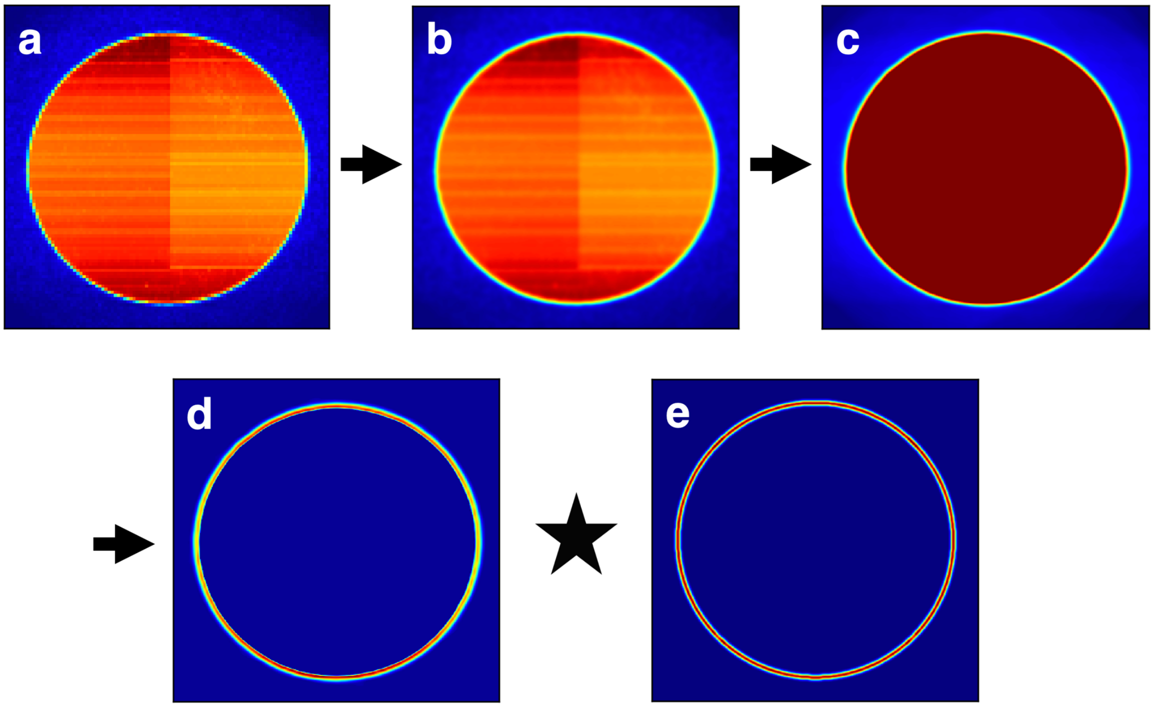

Supplementary Fig. 6 | Procedure for isolating long range effects on experimental diffraction data by central disk edge tracking. a, Diffraction pattern from the experimental data shown in Fig. 4 and Supplementary Fig. 1c,d, taken from a single probe position. For thin samples, the central disk is significantly more intense than the diffracted disks, allowing an edge to be defined. **b**, The raw diffraction patterns are upscaled using bilinear interpolation and filtered by a Gaussian function to reduce noise and smooth the edge of the disk. Some intensity variations within the disk remain, however. **c**, Counts above the minimum value within the central disk are therefore saturated to enable edge finding. **d**, The gradient of the saturated pattern is taken, and the magnitude of the gradient highlights the disk edge. The background values around the disk are also saturated here to decrease their contributions to later fitting. **e**, A circular pattern is generated with a radius equal to the central disk, centered on the average disk location. The pattern in **d** is then cross correlated with this pattern, and a two-dimensional Gaussian function is fit to the results, providing the position of disk rather than the features within it. This is performed at each probe position, generating a map of relative shifts of the disk. This process significantly suppresses contrast from short-range features while maintaining those from features appreciably larger than the probe.

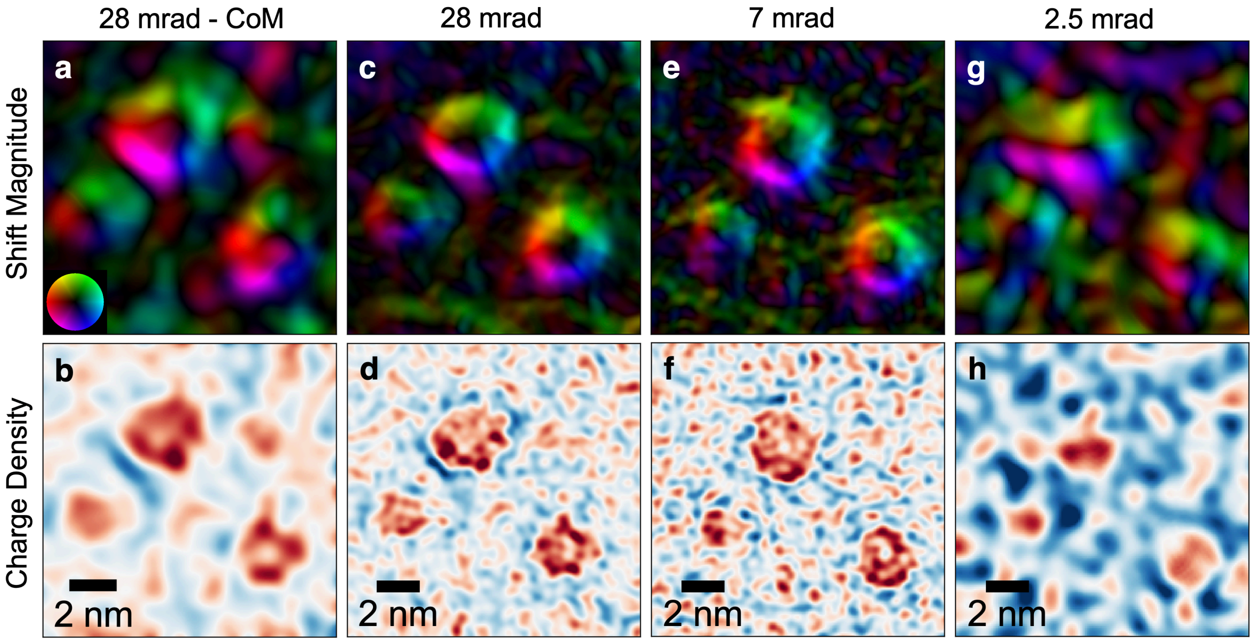

Supplementary Fig. 7 | Comparison of inverted CoM and disk edge tracking methods for mapping of long-range features. Performing a CoM measurement of the diffraction data at angles that include the edge of the central disk provides information about atomic-scale and longer-range features in the experimental data set shown in the main data. **a**, Application of a Gaussian filter approximately the size of the lattice spacing removes atomic-scale contrast while preserving longer-range information in the CoM signal and **b** corresponding information about charge density, as shown in Fig. 4. **c**, **d**, By performing disk edge tracking on the data instead of a CoM measurement, the atomic-scale contrast is entirely removed, but the long-range features are very similar (disk shift directions are inverted as CoM measurements are, due to the negative charge of the beam electrons). In addition, no spatial filter was applied here. **e**, **f**, Same as **c**, **d**, but with a semiconvergence angle four times smaller, at 7 mrad, with no spatial filter applied. **g**, **h**, Same as **c**, **d**, but with a semiconvergence angle more than an order of magnitude smaller, at 2.5 mrad, with no spatial filter applied. Each result qualitatively contains the same overall features, with decreasing resolution for smaller convergence angles, confirming these features arise mainly from shifts in the central disk, as expected for long-range fields.

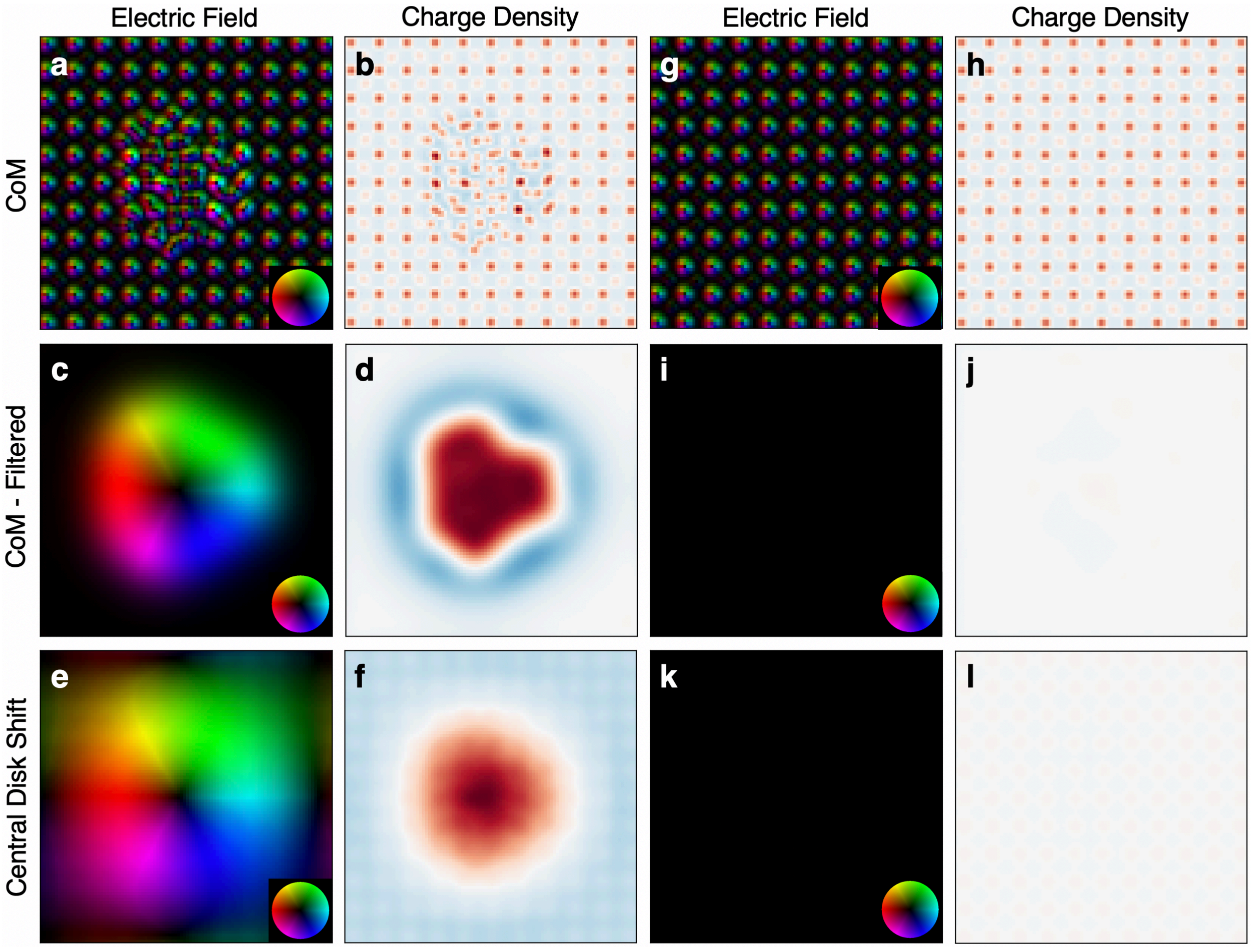

Supplementary Fig. 8 | Simulated inverted CoM and disk shift measurements on a Au particle-STO support system with no charge transfer included. a, Simulated inverted CoM map of the Au-STO structure used for the DFT calculations in Fig. 3, utilizing the independent atom model (no charge redistribution) and **b**, the corresponding atomic-scale "charge density" map. **c**, **d**, By applying a Gaussian filter analogous to Fig. 2, Fig. 4, and Supplementary Fig. 1, it is clear that longer-range features and a small positive "charge" is present on the particle even without charge transfer, due to the increased projected potential from the larger thickness. **e**, **f**, Disk shift tracking also produces long-range features, but with a significantly reduced intensity (maximum disk shift is 5x smaller than the maximum filtered CoM shift). In addition, the "charge" identified by disk shift tracking takes a different form than that from the filtered CoM signal, with a slow increase to the maximum at the particle, as opposed to the features from the filtered CoM, which are nearly flat across the particle. **g**–**l**, Identical to **a**–**f**, but with the Au particle removed, each panel normalized to the same value as its corresponding panel with the particle present. The altered structure of the support due to the particle has a negligible effect on both the CoM and disk shifts.

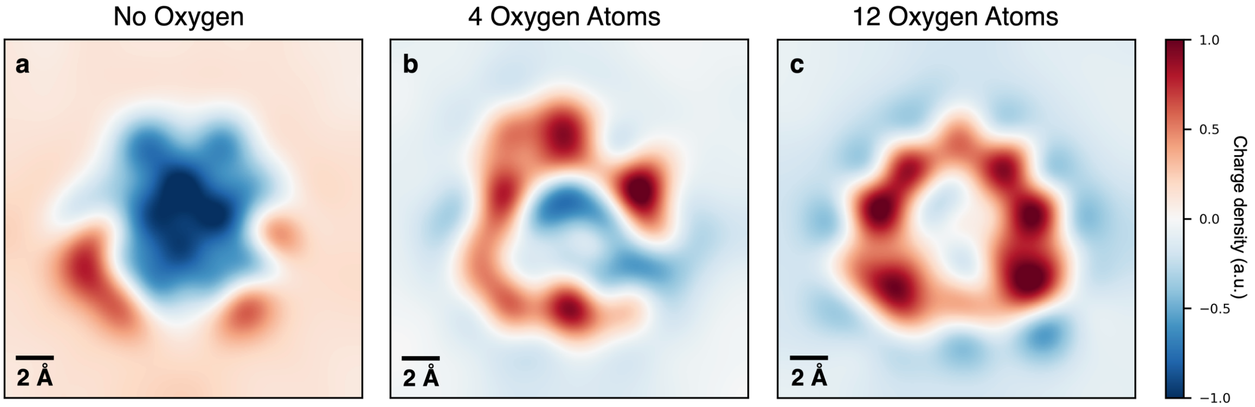

Supplementary Fig. 9 | Comparison of DFT calculation results for differing perimeter oxygen quantities, displayed top-down (along the (001) STO axis) as in the 4D-STEM experiments. **a**, Pristine Au particle on STO support, as shown in Fig. 3c, with a negative particle and positive surrounding support. **b**, Au particle with four oxygen atoms added to the perimeter, which induces an overall positive charge on the particle and a negative on the support. Most of the positive located near the particle perimeter, and in this case, the charge may not be uniform around the perimeter. **c**, Au particle with twelve oxygen atoms added to perimeter, which induces an overall positive charge on the particle and a negative in the support, with most of the positive located near the particle perimeter. In this case, the positive charge is more uniformly spread around the perimeter and extends further into the particle interior.

Supplementary References:

1. Cao, M. C. *et al.* Theory and practice of electron diffraction from single atoms and extended objects using an EMPAD. *Microscopy* **67**, i150–i161 (2017).
2. MacLaren, I. *et al.* On the origin of differential phase contrast at a locally charged and globally charge-compensated domain boundary in a polar-ordered material. *Ultramicroscopy* **154**, 57–63 (2015).
3. Müller-Caspary, K. *et al.* Measurement of atomic electric fields and charge densities from average momentum transfers using scanning transmission electron microscopy. *Ultramicroscopy* **178**, 62–80 (2017).
4. Krajnak, M., McGrouther, D., Maneuski, D., Shea, V. O. & McVitie, S. Pixelated detectors and improved efficiency for magnetic imaging in STEM differential phase contrast. *Ultramicroscopy* **165**, 42–50 (2016).
5. Haas, B., Rouvière, J.-L., Boureau, V., Berthier, R. & Cooper, D. Direct comparison of off-axis holography and differential phase contrast for the mapping of electric fields in semiconductors by transmission electron microscopy. *Ultramicroscopy* **198**, 58–72 (2019).
6. Wu, M. & Spiecker, E. Correlative micro-diffraction and differential phase contrast study of mean inner potential and subtle beam-specimen interaction. *Ultramicroscopy* **176**, 233–245 (2017).
7. Taplin, D. J., Shibata, N., Weyland, M. & Findlay, S. D. Low magnification differential phase contrast imaging of electric fields in crystals with fine electron probes. *Ultramicroscopy* **169**, 69–79 (2016).
8. Zweck, J., Schwarzhuber, F., Wild, J. & Galioit, V. On detector linearity and precision of beam shift detection for quantitative differential phase contrast applications. *Ultramicroscopy* **168**, 53–64 (2016).
9. Susi, T. *et al.* Efficient first principles simulation of electron scattering factors for transmission electron microscopy. *Ultramicroscopy* **197**, 16–22 (2019).
10. Lazić, I., Bosch, E. G. T. & Lazar, S. Phase contrast STEM for thin samples: Integrated differential phase contrast. *Ultramicroscopy* **160**, 265–280 (2016).
11. Silly, F. & Castell, M. R. Bimodal Growth of Au on $SrTiO_3$ (001). *Phys. Rev. Lett.* **96**, 86104 (2006).
12. Zhong, Z. & Hansmann, P. Tuning the work function in transition metal oxides and their heterostructures. *Phys. Rev. B* **93**, 235116 (2016).
13. Carroll, D. L., Wagner, M., Rühle, M. & Bonnell, D. A. Schottky-barrier formation at nanoscale metal-oxide interfaces. *Phys. Rev. B* **55**, 9792–9799 (1997).
14. Fu, Q. & Wagner, T. Interaction of nanostructured metal overlayers with oxide surfaces. *Surf. Sci. Rep.* **62**, 431–498 (2007).
15. Del Vitto, A., Pacchioni, G., Delbecq, F. & Sautet, P. Au Atoms and Dimers on the MgO(100) Surface: A DFT Study of Nucleation at Defects. *J. Phys. Chem. B* **109**, 8040–8048 (2005).
16. Sterrer, M. *et al.* Interaction of Gold Clusters with Color Centers on MgO(001) Films. *Angew. Chemie Int. Ed.* **45**, 2630–2632 (2006).
17. Sanchez, A. *et al.* When Gold Is Not Noble: Nanoscale Gold Catalysts. *J. Phys. Chem. A* **103**, 9573–9578 (1999).
